# Life Cycle and Genetic Identification of *Argas persicus* Infesting Domestic Fowl in Khyber Pakhtunkhwa, Pakistan

**DOI:** 10.3389/fvets.2021.664731

**Published:** 2021-05-19

**Authors:** Hafsa Zahid, Sebastián Muñoz-Leal, Muhammad Qayash Khan, Abdulaziz S. Alouffi, Marcelo B. Labruna, Abid Ali

**Affiliations:** ^1^Department of Zoology, Abdul Wali Khan University Mardan, Mardan, Pakistan; ^2^Departamento de Patología y Medicina Preventiva, Facultad de Ciencias Veterinarias, Universidad de Concepción, Concepción, Chile; ^3^King Abdulaziz City for Science and Technology, Riyadh, Saudi Arabia; ^4^Departamento de Medicina Veterinaria Preventiva e Saúde Animal, Faculdade de Medicina Veterinaria e Zootecnia, Universidade de São Paulo, São Paulo, Brazil

**Keywords:** soft ticks, life cycle, *Argas persicus*, domestic fowls, Pakistan

## Abstract

Ticks transmit numerous pathogens to animals including humans; therefore, they are parasites of health concern. Soft ticks infesting domestic fowl in Pakistan are carriers of viruses and bacteria and cause unestimated economic losses in the poultry sector. The current study was intended to identify soft ticks infesting domestic fowl and understand their spatiotemporal distribution along 1 year. A sum of 7,219 soft ticks were collected from 608 domestic fowl in 58 infested shelters; 938 (12.9%) ticks were found on the host and 6,281 (87%) in the shelters. The collected ticks comprised 3,503 (48.52%) adults including 1,547 (21.42%) males and 1,956 (27.09%) females, 3,238 (44.85%) nymphs, and 478 (6.62%) larvae. The most prevalent life stages were adults, followed by nymphs and larvae. Overall tick prevalence considering all visited shelters was 38.66% (58/150). The highest tick prevalence was found in district Lakki Marwat (50.03%) followed by Peshawar (31.08%) and Chitral (18.88%) districts. All ticks were morpho-taxonomically identified as *Argas persicus*. To determine their life cycle, adult *A. persicus* were reared in the laboratory infesting domestic fowl (*Gallus gallus domesticus*). The life cycle was completed in 113–132 days (egg to egg) with a mean temperature of 33 ± 3°C and relative humidity of 65 ± 5%. Individual ticks were used for DNA extraction and subjected to polymerase chain reaction (PCR) using specific primers for the amplification of a partial fragment of mitochondrial cytochrome oxidase subunit I (*cox1*) and 16S ribosomal RNA (16S rRNA) genes. Obtained amplicons were compared using basic local alignment search tool (BLAST) to scan for homologous sequences. Phylogenetic trees showed *A. persicus* from Pakistan clustering with conspecific sequences reported from Australia, Chile, China, Kenya, and the United States. This is the first study aiming to reproduce the life cycle of *A. persicus* and genetically identify this tick in the region. Further studies are encouraged to investigate the pathogens associated with this soft tick species in Pakistan.

## Introduction

Soft ticks in the genus *Argas* are parasites associated mostly with birds. Sixty-one species are currently described in the genus (1, 2), and at least four are parasites of fowl, namely, *Argas miniatus, Argas persicus, Argas radiatus*, and *Argas sanchezi* (3). *Argas persicus*, commonly referred as the fowl tick, is distributed chiefly in all continents (3). Wide analyses of the geographic distribution of *A. persicus* in the American Continent pointed out that this soft tick occurs in dry and subtropical environments and be absent in tropical latitudes (4).

Pakistan is immersed in one of the subtropical regions of the world. Suitable temperature and humidity conditions in the country facilitate the growth and development of ticks in domestic animals. The geography and climatic patterns of Pakistan covaries from high altitude and cold environments (Himalaya Mountains) to low and warm lands toward the sea. Soft ticks of the genus *Argas* in Pakistan are currently represented by four species only: *A. persicus, Argas reflexus, Argas abdussalami*, and *Argas rousetti* (1, 5–7). As in many other geographic regions of the world, *A. persicus* is a frequent fowl parasite in Pakistan (5, 6, 8).

Various environmental conditions have been reported showing an innocuous effect on tick diversity (9). In accordance with other subtropical regions of the world, the tick fauna of Pakistan concentrates within several regional climatic zones, especially in arid, sub-arid, and humid areas (5, 6, 10). Environmental conditions such as host availability, precipitation, temperature, and humidity shape the life cycle of a tick species (11, 12). As in other Argasids, when unfavorable environmental conditions prevail, *Argas* spp. can starve for several years sheltered in crevices or cracks (1). These ticks have the ability to reduce dehydration and enter diapause periods (13–15). Larvae are slow feeders generally and stay attached to their host for 5–10 days (13). Each life stage requires a successful and short blood uptake to molt (13). Nymphs feed several times and molt until reaching maturity as males or females (14, 16).

Pakistan has the 11th largest poultry industry in the world with a production of 1,163 million broilers annually. The poultry sector provides employment to over 1.5 million people, and investment is more than Rs 700 billion currently (Pakistan Economic Survey 2019–2020, Ministry of Finance, Government of Pakistan). Although large-scale poultry production grows in the country, domestic fowl are still abundant in cities and rural areas and bring important economic benefits to the population. Domestic fowl are typically raised either inside hen houses or freely, congregating at night, a fact that favors the maintenance of nidicolous *Argas* spp. (1). As blood sucking parasites, *Argas* spp. impose stress on their avian hosts, therefore affecting their health. Despite the economic importance, studies on ticks associated with fowl have been neglected in Pakistan. The present study aimed to assess the distribution, life cycle, and phylogenetic position of *Argas* ticks infesting domestic fowl along different regions of north western Pakistan.

## Materials and Methods

### Study Sites

**Figure 1:**
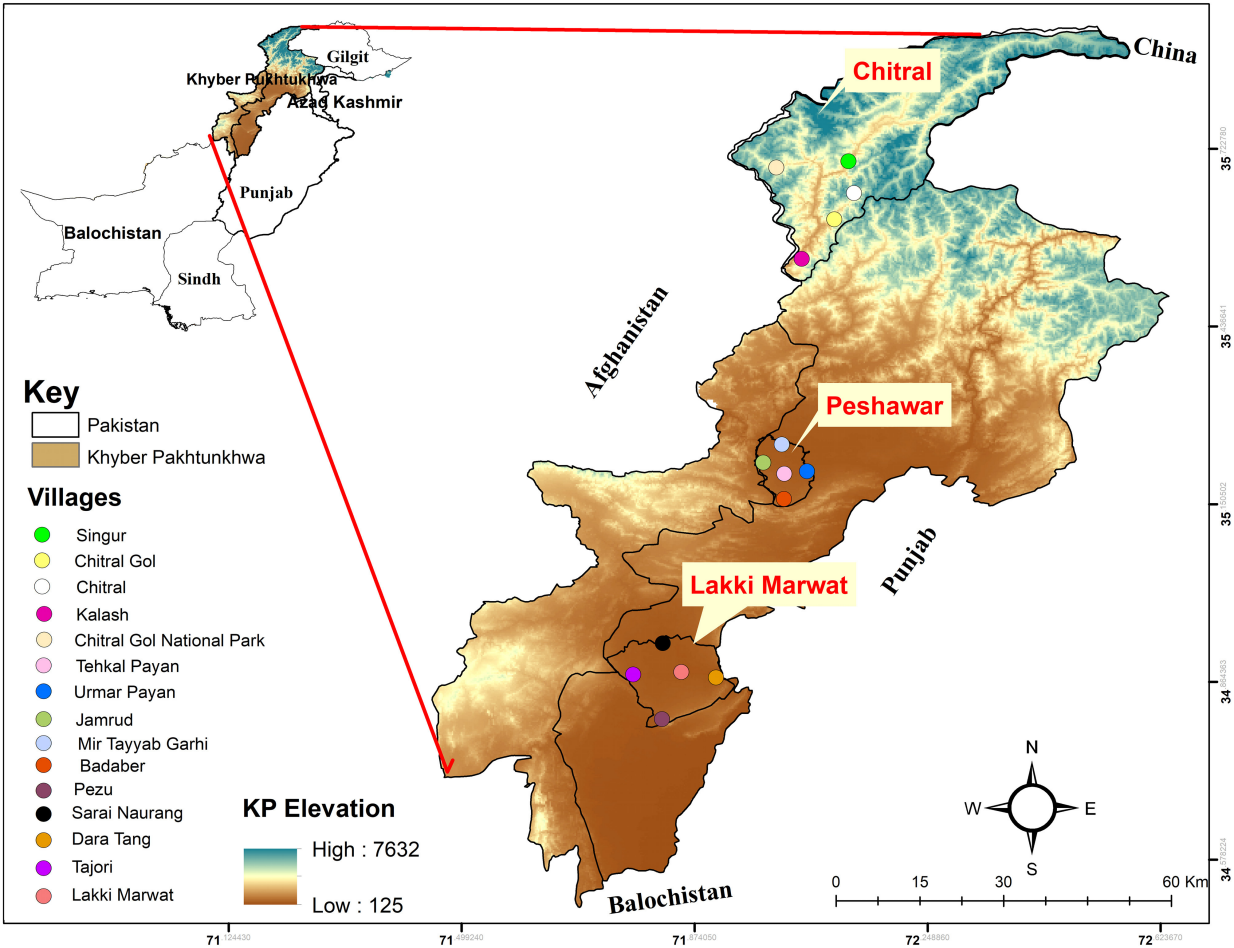
Elevation map showing the study area where *Argas* ticks were collected.

Tick collection was carried out during April 2018 to March 2019 in the region of Khyber Pakhtunkhwa (KP), specifically in the district of Peshawar (34°01′36.2″ N; 71°31′47.4″E, 331 m), Chitral (35°53′40.9″N; 71°41′31.1″E, 1,494 m), and Lakki Marwat (32.6135°N; 70.9012°E, 255 m). The Global Positioning System was used to collect the exact coordinates of each location, and a map was designed using ArcGIS v 10.3.1 (Figure 1).

The KP province is located in the north western region of Pakistan. Peshawar, the capital city, extends along the wide-ranging valley of Peshawar rivers. The winter begins in mid-November and ends in March, with a mean temperature ranging between 4 and 18.35°C. The summer starts in April and ends in October, with a mean temperature ranging from 25 to 40°C. Rainfall occurs both in winter and summer (climate-data.org). The selected areas for sampling in Peshawar district included Badaber villages, Jamrud, Mir Tayab Garhi, Peshawar, and Umar Payan. The largest district, Chitral, is situated in the north of KP province with an area of 14,850 km^2^ and has a Mediterranean climate with warm summers and mild winters. The mean temperature during summer ranges between 22 and 32°C from April until October. The mean temperature during winter ranges from 0 to 11.4°C during November to March. Rainfall occurs throughout the year (climate-data.org). Areas selected for tick collection in district Chitral were Chitral, Chitral Gol National Park, Gahirat Gol, Kalash, and Singur. The district Lakki Marwat has desertic lands with abundant sand dunes and dry and hot weather. Summer starts in April and ends in late October, with the hottest month in June (30–45°C). A moderate winter starts in early November and continues until March, with temperatures ranging between 4 and 20°C. Rainfall is rare and mostly occurs in July and August (climate-data.org). The areas selected for sample collection in district Lakki Marwat included Lakki Marwat, Dara Tang, Pezu, Sarai Naurang, and Tajori.

### Collection of Ticks, Prevalence, and Mean Infestation

A total of 150 shelters were visited twice per month along a year. In each of them, birds, wall cracks, ceilings, and floors were carefully examined in the search for soft ticks. The bark of trees in rural areas where the domestic fowl used to rest or shelter was examined as well. Ticks were collected alive in labeled sterile plastic bottles and manipulated using fine tweezers to avoid damaging the specimens. Collected ticks were transported to the Department of Zoology, Abdul Wali Khan University, Mardan, for identification and further analyses. The collected larvae and all nymphal instars were preserved in 100% ethanol.

The prevalence and mean infestation of ticks were calculated using the following formula:

$$\text{Shelter infestation prevalence }(\%)=\frac{\text{No}.\text{ of infested shelters}}{\text{Total no}.\text{ of shelters visited}}\times 100$$

$$\text{Host mean infestation}=\frac{\text{Total no}.\text{ of ticks on host}}{\text{No}.\text{ of infested host}}$$

### Morphological and Molecular Identification of Ticks

Ticks were rinsed with distilled water and 70% ethanol for the removal of surface contamination. For nymphs and adults, external morphology was observed using a stereomicroscope (HT Stereozoom) and compared with available taxonomic keys (3, 14). Special attention was put to the anatomy of the idiosomal margins, since the integumental cell-like structures that this body region exhibits are important to separate species in the genus *Argas* (3). Identified engorged females were kept in Petri dishes at room temperature for life cycle observations.

Morphological diagnoses were confirmed by molecular tools. For that purpose, field collected specimens (one female, one male, one nymph, and two pools of 10 larvae per locality) were submitted to DNA extraction. Ticks were perforated with a sterile needle inside 1.5-ml tubes and heated at 50°C for ethanol evaporation. Genomic DNA was extracted using GeneJET Genomic DNA Purification Kit (Thermo Fisher Scientific, Waltham, MA, United States) following the manufacturer DNA extraction protocol. The integrity of DNA was confirmed by gel electrophoresis, and DNA concentration was quantified using a Nanodrop ND-100 (Thermo Fisher Scientific, Waltham, MA, United States). Samples were stored at −20°C.

PCR was performed to amplify two mitochondrial markers, a 606-base-pair (bp) fragment of the *cox1* gene and a 240-bp fragment of the 16S ribosomal RNA (rRNA) gene. In particular, for the 16S rRNA gene, we downloaded 82 homolog sequences from *Argas* spp. available in National Center for Biotechnology Information (NCBI). Based on an alignment of these sequences, primers 3′-TTTGGGACAAGAAGACCCTATGAA TTT-5′ (forward) and 3′-ACATCGAGGTCGCAATCAATTTTATC-5′ (reverse) were designed using highly conserved regions detected with Vector NTI v 11.5.3. Primers GGAGGATTTGGAAATTGATTAGTTCC-5′ (forward) and 3′-ACTGTAA ATATATGATGAGCTCA-5′ (reverse) were employed to amplify *cox1* gene (17). PCR was performed in a 20-μl mix [1 μl of forward primer, 1 μl reverse primer, 2 μl of template DNA, 12 μl Dream*Taq* PCR Master Mix (2×) (Thermo Scientific, Waltham, MA, United States), and 4 μl PCR grade water]. The PCR conditions were set as follows: initial denaturation temperature of 95°C for 5 min, followed by 35 cycles of 95°C denaturation for 30 s, 53°C (*cox1*) and 56°C (16S rRNA); annealing for 30 s, 72°C extension for 1 min; and final extension of 72°C for 5 min. PCR products were run on ethidium bromide-stained agarose gels and observed by UV trans-illumination (UVP BioDoc-It Imaging System, Upland, CA, USA).

Expected size amplicons were sequenced at Macrogen, Korea. The generated sequences were trimmed and assembled in SeqMan v 5.00 (DNAstar). A BLAST analysis was performed using the obtained consensuses (18). An alignment for each sequenced gene was constructed with ClustalW and edited in BioEdit alignment editor V 7.0.5 (19). Phylogenetic analyses were inferred by the maximum likelihood method for both genes using PhyML (20), with the General Time Reversible (GTR) model, five substitution rate categories, and 1,000 bootstrap replicates.

### Life Cycle

A subgroup of five engorged females per collection site was separated to investigate the life cycle of *A. persicus*. Ticks were kept inside an incubator at 33 ± 3°C and 65 ± 5% relative humidity for survival and oviposition. Laid eggs were carefully transferred to 5-ml sterile plastic syringes and sealed with wet cotton to provide humidity. Hatched larvae, subsequent nymphal instars, and adults were feed on domestic fowl (*Gallus gallus domesticus*).

### Statistical Analysis

All recorded observations such as collection data and life cycle were assembled and arranged in the spreadsheets of Microsoft Excel V 2013 for descriptive analysis [mean and standard deviation (SD)]. Chi-square test was used for chi-square difference (χ^2^) using the Statistical Package for the Social Sciences (IBM SPSS, Version 21) considering 95% confidence interval (CI) and a significant *P* < 0.05.

### Ethical Approval

The current study was approved by the advance studies and research board (Dir/A&R/AWKUM/2020.4871) of the Abdul Wali Khan University, Mardan. A written or oral consent was taken during collection from the owner of domestic fowl.

## Results

### Collected Ticks

**Figure 2:**
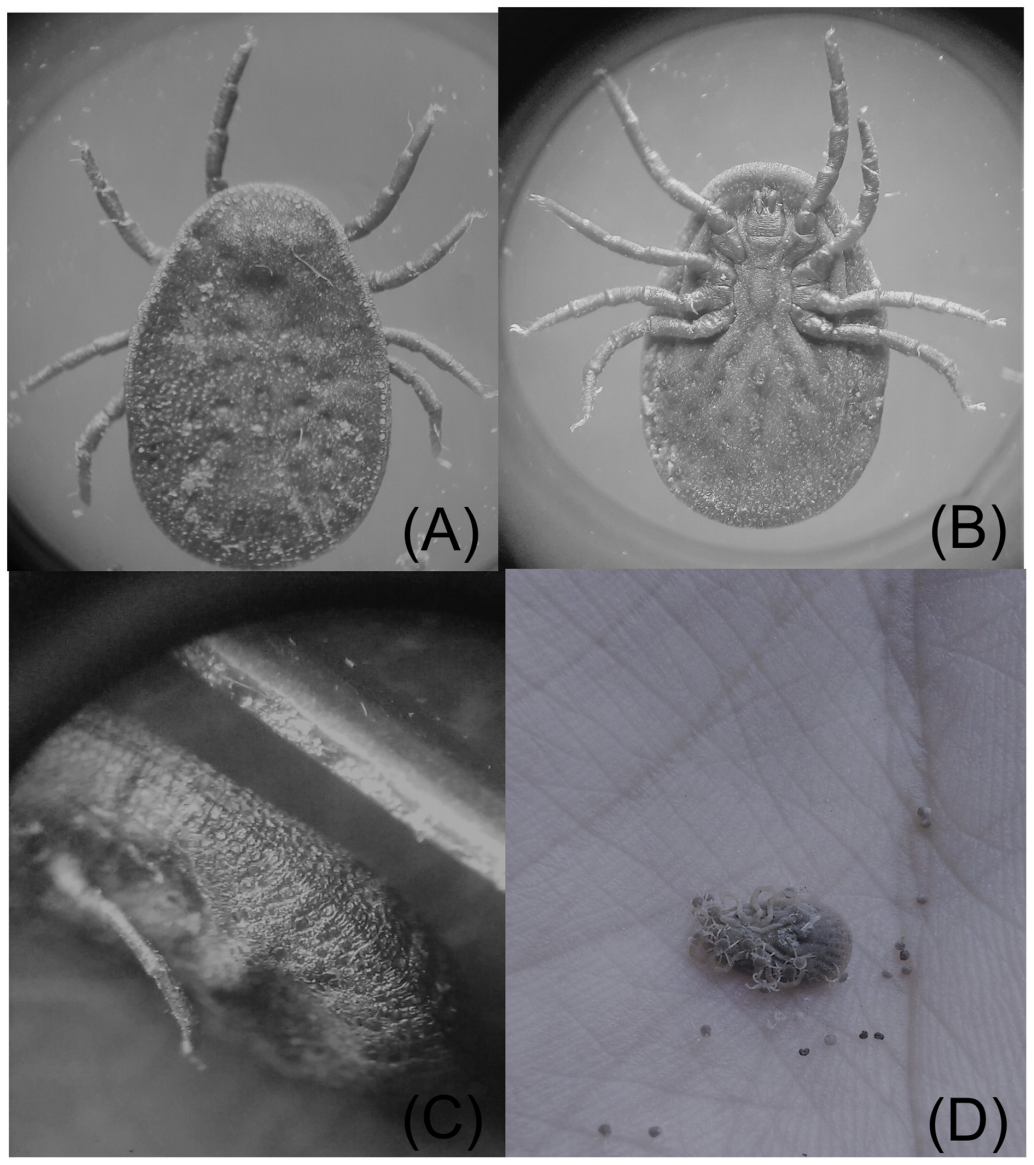
**(A)** Dorsal, **(B)** ventral, and **(C)** lateral view of female *A. persicus*. **(D)** Maternal-like behavior in *A. persicus*.

A total of 7,219 *Argas* ticks were collected from 608 domestic fowl in 58 infested shelters. Among these, 3,612 (50.03%) were collected in district Lakki Marwat, 2,244 (31.08%) in Peshawar, and 1,363 (18.88%) in Chitral. Overall tick prevalence considering all visited shelters was 38.66% (58/150). Different life stages were collected, including 478 (6.62%) larvae, 3,238 (44.85%) nymphs, 1,547 (21.42%) males, and 1,956 (27.09%) females (Table 1). A total of 938 (12.9%) ticks were found feeding on domestic fowl, while 6,281 (87%) were collected in the shelters and crevices. In particular, 373 larvae (78.03%) were found on domestic fowl, and 105 (21.96%) were collected wandering in the shelters. Postlarval stages were more abundant in shelters than on birds (Table 2). All nymphs and adult ticks were identified morphologically as *A. persicus* because of having <100 integumental cells around the body margin and by the presence of a lateral line (Figures 2A–C) (3). Pools of larvae were identified by molecular tools.

**Table 1:** The abundance of different stages of collected *Argas persicus* ticks.

**District**	**Total (%)**	**Larvae (%)**	**Nymph (%)**	**Male (%)**	**Female (%)**	***X***^**2**^	***P*-value**
Chitral	1,363 (19)	87 (6)	746 (58)	204 (15)	326 (24)		
Peshawar	2,244 (31)	174 (6)	1,016 (45)	452 (20)	602 (27)	102.7	0.001
Lakki marwat	3,612 (50)	217 (8)	1,476 (40)	891 (24)	1,028 (28)		
Total	7,219 (100)	478 (7)	3,238 (45)	1,547 (21)	1,956 (27)		
Mean	2,406	159.3	1,079	515.7	652.0		
SD	1,133	66.23	369.1	347.9	353.7		
CI	0–324	162–199	0–138	0–153	0–522		

**Table 2:** Number of collected *Argas persicus* ticks according to domestic fowl hosts (H) and their shelters (S).

**District**	**Larvae H/S**	**Nymph H/S**	**Male H/S**	**Female H/S**	**Total H/S**
Chitral	78/9	103/643	11/193	16/310	208/1,155
Peshawar	123/51	153/863	17/435	23/579	316/1,928
Lakki marwat	172/45	146/1,330	43/848	53/975	414/3,198
Total	373/105	402/2,836	71/1,476	92/1,864	938/6,281
Percentage	78.03/21.96	12.41/87.58	4.58/95.41	4.70/95.29	12.9/87

### Spatiotemporal Distribution of Ticks, Prevalence in Shelters, and Host Mean Infestation

**Figure 3:**
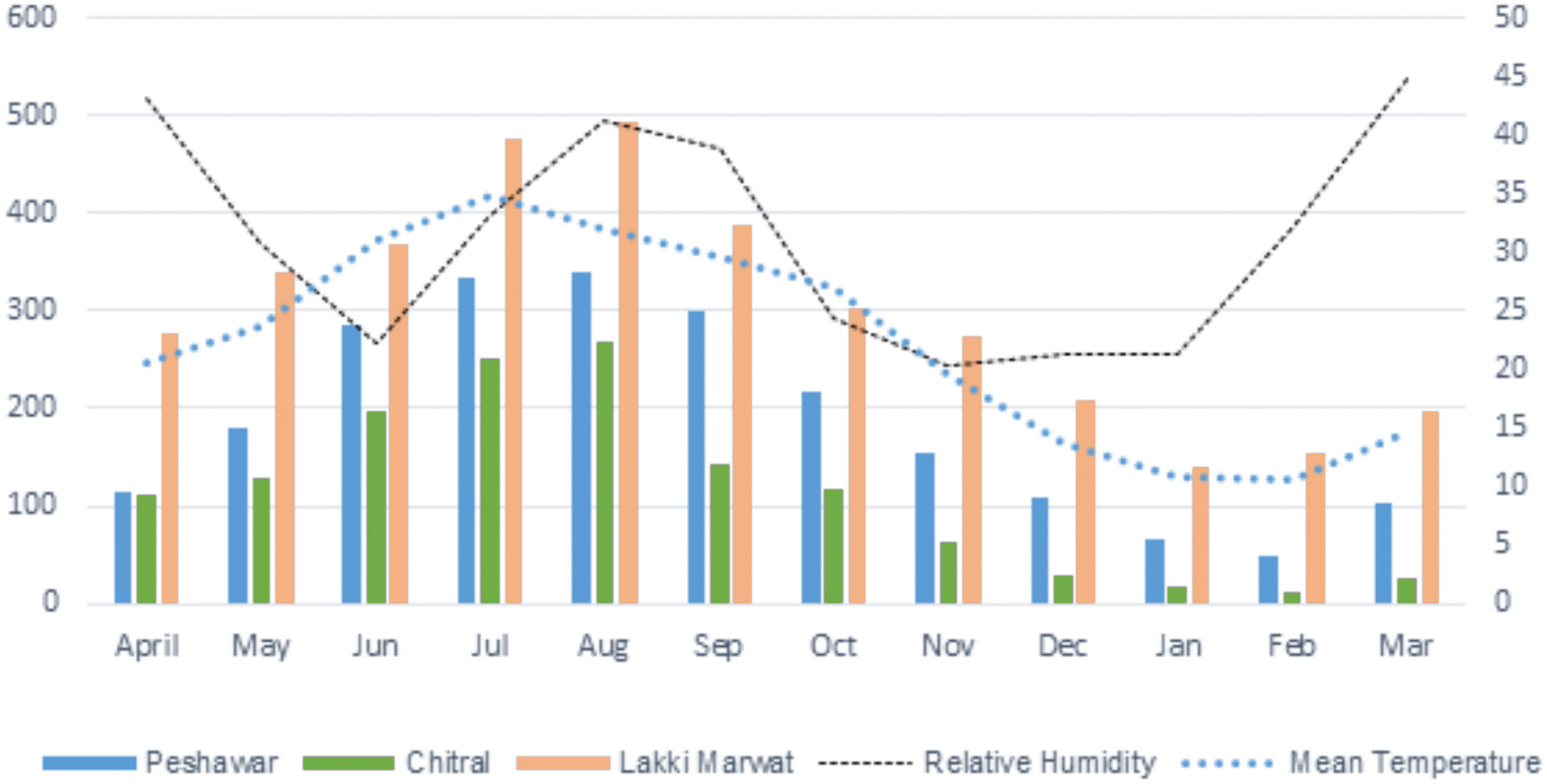
Chart showing spatial distribution of *Argas* ticks in selected districts of KP.

The highest tick infestation was found during July, while the lowest tick infestation was observed in the month of January followed by February and December (Table 3, Figure 3). Considering the collection site, the highest prevalence was found in Lakki Marwat where a total of 3,612 ticks were collected from 269 domestic fowl followed by Peshawar where 2,244 ticks were collected from 197 domestic fowl, and the least prevalence was found in Chitral where 1,363 ticks were found on 142 domestic fowl. Nymphal instars (3,238; 45%) were most abundant, followed by adult females (1,956; 27%) and males (1,547; 21%). On the other hand, larval stages comprised 478 (7%) individuals in all three selected districts of KP (Table 1).

**Table 3:** District wise spatial distribution of infested hosts (domestic fowl) and collected ticks (*Argas persicus*).

	**No. of infested host**	**No. of ticks collected**	**No. of infested host**	**No. of ticks collected**	**No. of infested host**	**No. of ticks collected**
Total (%)	197 (32.40%)	2,244 (31.08%)	142 (23.35%)	1,363 (18.88%)	269 (44.24%)	3,612 (50.03%)
Mean	39.4	448.8	28.4	272.6	53.8	722.4
SD	12.68	104.4	8.649	72.98	8.468	109.3
CI	23.6–55.1	319–578	17.66–39.14	182–363	43.2–64.3	586–858
*P*-value	0.0006		0.0011		0.0001	

The hot climatic district Lakki Marwat, where the average temperature during summer ranges between 30 and 45°C, had the most infested shelters (26/50, 52%) followed by the moderate temperature (25–40°C) district Peshawar (18/50; 36%) and low temperature (22–32°C) district Chitral (14/50; 28%). On-host, tick infestation was calculated in each shelter, and the overall mean infestation on a single host was 11.87. The average tick infestation on domestic fowl in Lakki Marwat, Peshawar, and Chitral was 13.42, 11.39, and 9.59, respectively.

### Life Cycle

#### Egg Laying and Larval Hatching

We did not observe mating of *A. persicus* on the host body and was observed off-host. Female ticks were observed to feed on domestic fowl for 30–35 min and laid a batch of (20–30) rounded pale-yellow eggs after 12 ± 3 days. Successive feedings and ovipositions were observed up to six to seven times until the female died. After 2–5 days, the eggs became dark and dry and hatched after 15–20 days of incubation (Table 4). The body measurement of *A. persicus* was taken at each stage (Table 5). The emerged larvae were found to remain on the female’s ventral surface (Figure 2D). After 5 ± 2 days, the larvae started questing for hosts and were allowed to feed on domestic fowl. Overall, the larvae remained attached mostly under the wings of the birds and for a period of 5 ± 1 days (Table 4).

**Table 4:** Durations of life stages of *Argas persicus* infesting natural host (domestic fowl).

**S#**	**Traits**	**Time duration of life stage (mean ± SD)**	No. of examined ticks^**^
1	Pre-oviposition	12 ± 2.5 days	5
2	Eggs laying	2 ± 0.7 days	5
3	Eggs incubation	17 ± 1.3 days	–
4	Larvae free living	5 ± 1.6 days	86
5	Larvae attachment on host	5 ± 1.3 days	15
6	Larvae molting to nymph	12 ± 1.8 days	12
7	^*^Nymph feeding	22 ± 1.6 min	–
8	Nymph molting	12 ± 1.9 days	12
9	Adult feeding	33 ± 1.8 min	12
10	Complete life cycle	113–132 days (egg to egg)	–

* *Nymphal stage includes a series of five nymphal instars*.

** *The number of ticks were same for three selected districts*.

**Table 5:** Body measurements of *Argas persicus*.

Ticks life stages^*^	**Average weight in g**	**Size in mm (mean ± SD)**
Male (UF)	0.003011	5.12 ± 0.12
Male (F)	0.010847	5.55 ± 0.55
Female (UF)	0.010833	6.25 ± 0.25
Female (F)	0.0325	8.12 ± 0.120
Female (FAO)	0.012375	8.12 ± 0.12
Egg (100×)	0.000099 (0.0099)	–
Larvae (F)	0.000133	1.001 ± 0.2
1st Nymph	0.0001	1.35 ± 0.15
2nd Nymph (UF)	0.0002	1.34 ± 0.08
2nd Nymph (F)	0.0005	2.05 ± 0.2
3rd Nymph (UF)	0.0008	2.02±-0.2
3rd Nymph (F)	0.0013	2.70 ± 0.07
4th Nymph (UF)	0.003	2.71 ± 0.06
4th Nymph (F)	0.004	3.72 ± 0.03
5th Nymph (UF)	0.007543	3.73 ± 0.02
5th Nymph (F)	0.014285	5.04 ± 0.03

*F, fed; UF, unfed; FAO, fed after oviposition*.

* *The average of five tick’s weight is given in the table*.

#### Nymphal Instars

The larval stage was found to molt to eight-legged nymphs after 12 ± 3 days. We noted a total of five nymphal instars, each one feeding for 15–20 min. The specimens molted to the next nymphal instar in 12 ± 3 days after feeding.

#### Adults

Female ticks emerged from the fourth and fifth nymphal instars, while the third and fourth nymphal instars mostly molted to male ticks. We observed a preoviposition period of 12 ± 3 days in the incubator.

### Genetic Identification and Phylogenetic Analysis

**Figure 4:**
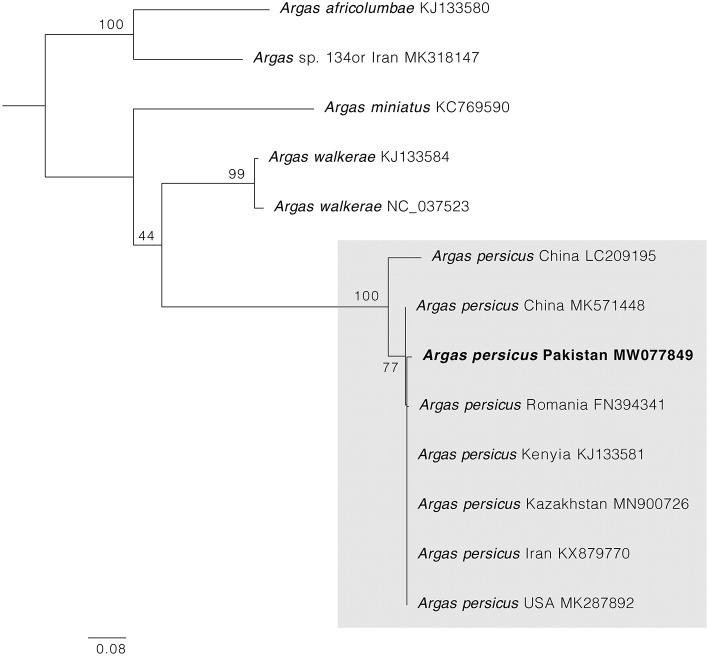
Maximum likelihood tree constructed for the *cox1* sequence of *A. persicus* generated in this study. Species names are followed by country and accession numbers in parentheses. Bootstrapping values (1,000) are shown at each branch. The bar represents 0.08 substitutions per site. Sequence obtained in the present study is highlighted in bold.

**Figure 5:**
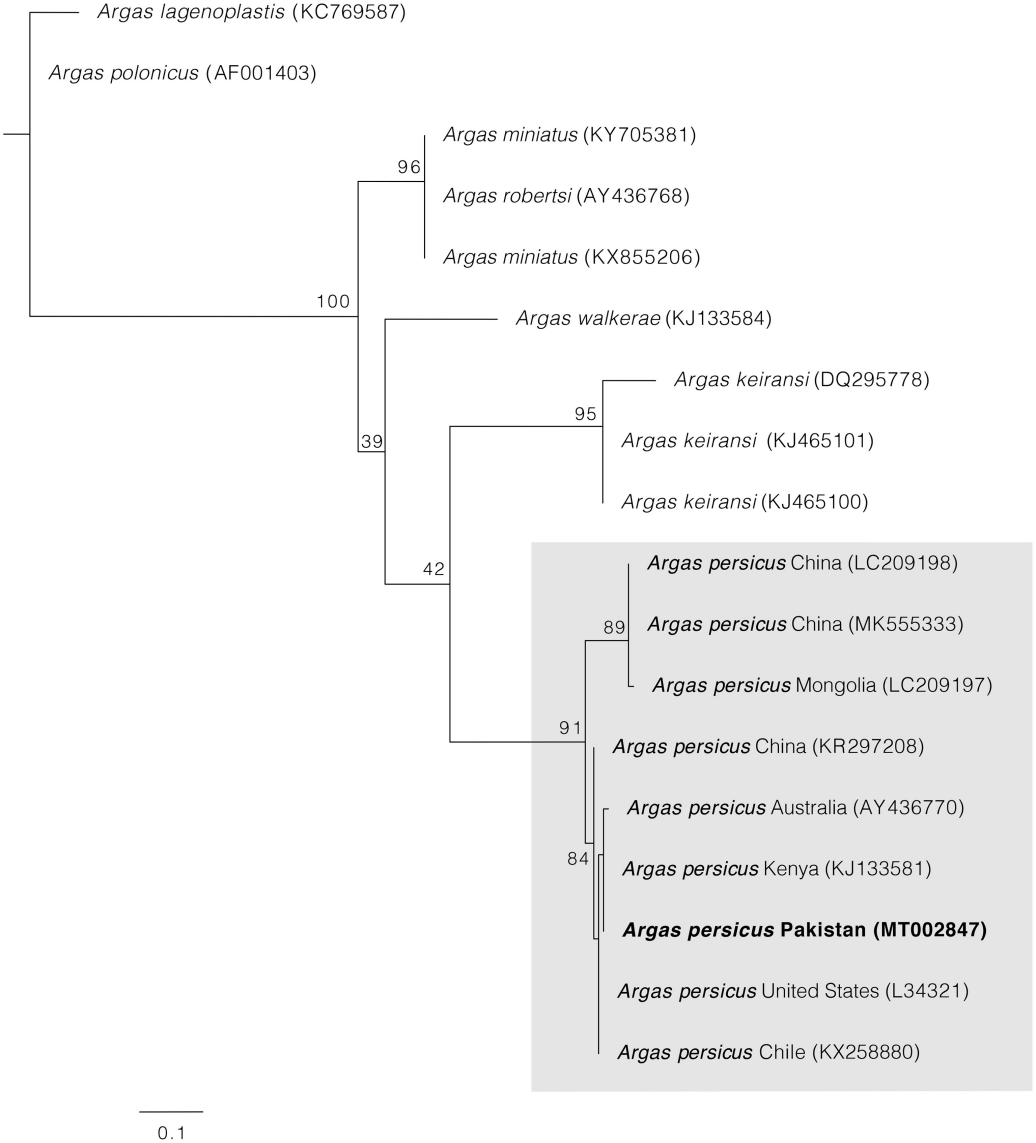
Maximum likelihood tree constructed for the 16S sequence of *A. persicus* generate in this study. Species names are followed by country and accession numbers in parentheses. Bootstrapping values (1,000) are shown at each node. The bar represents 0.1 substitutions per site. Sequence obtained in the present study is highlighted in bold.

PCRs for *cox1* and 16S rRNA mitochondrial genes were positive in all samples, and one single haplotype for each gene was obtained. BLAST comparisons confirmed our morphological diagnosis since both *cox1* and 16S rRNA genes showed 98–100% of identity with homologous sequences of *A. persicus* from other regions of the world. The phylogenetic analysis for *cox1* gene showed *A. persicus* from Pakistan clustering with conspecific sequences of China, Iran, Kazakhstan, Kenya, Romania, and the United States. In the 16S rRNA gene phylogenetic tree, the sequence from Pakistan was grouped with *A. persicus* from Australia, Chile, China, Kenya, and the United States (Figures 4, 5). GenBank accession numbers for the sequences generated in this study are MW077849 and MT002847.

## Discussion

The majority of studies on ticks has been focused on the Ixodidae family (hard ticks), their associated pathogens, and risks for public and veterinary health (5, 6, 21–26). On the other hand, despite the economic importance of poultry, studies on soft ticks infesting domestic fowl have been neglected. In Pakistan, studies have been focused on the hard tick fauna, associated risk factors, morphological and molecular identification, seasonal variations, phylogenetic, and pathogens they carry; however, studies on soft ticks are limited (5, 6, 27). The present study reports the spatiotemporal distribution and molecular characterization of *A. persicus* infesting domestic fowl in various regions of KP, Pakistan, for the first time. The constructed phylogenetic tree revealed an evolutionary relationship of herein collected *A. persicus* with *Argas* ticks from Romania, Australia, China, Chile, Iran, Kazakhstan, Kenya, and United States. Furthermore, the life cycle of *A. persicus* was carefully documented for the first time in the region under laboratory conditions using domestic fowl as hosts. Overall, the period in which *A. persicus* completes its life cycle was studied for the first time in the region.

*A. persicus* has adapted to parasitize fowl worldwide (28–31), and in the present study, we found that domestic fowl of the selected districts were highly infested by this soft tick. Extremely elevated and low temperatures in the northern regions of KP, especially in district Chitral, might serve as less-favorable environmental conditions for the survival of *A. persicus*, a fact that has been observed in other parts of the world for soft ticks (32). Tick survival and prevalence mostly depend on the environmental conditions such as temperature and humidity (11, 12). Districts with moderate climate such as Peshawar and western regions (Lakki Marwat) were found favorable for the infestation by *A. persicus*. It is well-known that high temperature and high humidity favor the development and persistence of various ticks, including *A. persicus*, in tropical and subtropical distributions (6, 30, 32, 33). The spatiotemporal distribution of *A. persicus* in different temperature regimes agrees with our previous report (6).

As the survival of the tick requires favorable temperature and humidity, the life span of the tick may differ from one region to another (11, 12). The life cycle of *A. persicus* was studied and documented in detail to obtain biological evidence for effective control strategies. Knowing the life cycle of a tick species is important because each life stage can vary in terms of pathogen transmission to the host (34–37). Larval stages are the most suitable life stage of a tick for the use of acaricides or vaccine (to date there is no available vaccine for the control of soft ticks) on the host because it remains attached for days on the host and can be easily targeted. In contrast, nymphal, and adult ticks feed for a short period of time and mostly remain off-host sheltering in the environment, a fact that precludes effective control measures.

Studies on the life cycle of *A. persicus* have been performed in populations of Egypt (16), and three nymphal instars were observed. Walker et al. (14) reported that, in general, there may be four nymphal instars in the life cycle of *A. persicus*. The findings of our study slightly disagree with previous reports in that five successive and prominent nymphal instars were observed before the adult stage. Differences in the number of nymphal instars and life span observed in our study with respect to Walker et al. (14) and El-Kammah and Abdel-Wahab (16) may be due to the difference in host and environmental conditions.

We observed that the *A. persicus* adult females kept their larvae restricted to the ventral surface likely providing protection, a behavior that could be interpreted as maternal care. The same phenomenon has been previously observed in other soft ticks of the genus *Argas* and *Antricola*, such as *Argas striatus, Argas transgariepinus*, and *Antricola marginatus* (38, 39).

Many soft ticks are morphologically similar and lead to misidentification up to the species level based on external morphology; therefore, mistakes in their identification are not uncommon and have been described previously (40, 41). Genetic data are often required to accurately identify a given soft tick species. Indeed, mitochondrial genes such *cox1* and 16S rRNA have been utilized as markers for molecular identification of various tick species including soft ticks (42–44). Since the current systematics of soft ticks is still controversial, we opted to use the *cox1* and 16S rRNA to explore the phylogenetic relationships of *A. persicus* ticks from KP, Pakistan. The generated sequences showed 100% similarity to each other obtained from different regions in KP, Pakistan, and these generated sequences showed the closest similarities (98–100%) to the GenBank sequences deposited from various regions of the world. This fact indicates that the sequences for *the cox1* and 16S rRNA genes of *A. persicus* are highly conserved, even between vastly distanced populations. The findings of the present study are in agreement with previous reports, which suggest the use of *the cox1* and 16S rRNA genes as a suitable marker to identify *A*. *persicus* (4, 45–47). In the phylogenetic analyses, the generated sequence clustered in a separate subclade with the sequences deposited in GenBank for *A. persicus* from Australia, China, Chile, Kenya, and the United States. On the other hand, some close phylogenetic relationship was confirmed between different *Argas* species based on *cox1* and 16S rRNA, for instance, *Argas robertsi* from Australia and *Argas miniatus* from Brazil (47). These findings evidenced that there is a close phylogenetic relationship between *Argas* species from different geographic regions that deserves further attention. This may also be due to the lack of sufficient data deposited in NCBI from various regions for *Argas* ticks. Therefore, the addition of generated sequences during the present study is essential for drawing the evolutionary analysis of soft ticks.

## Conclusion

The present study reported for the first time *A. persicus* ticks infesting domestic fowl in three districts, including the moderate temperature-region Peshawar, cold climatic-region Chitral, and hot climate-region Lakki Marwat, in KP, Pakistan. The ticks collected from the hosts were fewer in number compared to specimens found in the shelters. The life cycle of *A. persicus* in natural conditions was investigated for the first time in the region, and five nymphal instars were observed before the emergence of adults. The presence of *A. persicus* was confirmed at the molecular level by using *cox1* and 16S rRNA genes, and in phylogenetic trees, the generated sequences clustered with sequences from Australia, Chile, China, Kenya, and United States. Future studies are encouraged to investigate *A. persicus* as a potential reservoir for pathogens affecting the poultry industry and causing known and unknown infections and economic losses.

## Data Availability Statement

The datasets presented in this study can be found in online repositories. The names of the repository/repositories and accession number(s) (MW077849 and MT002847) can be found in the article.

## Ethics Statement

The current study was approved by the advance studies and research board (Dir/A&R/AWKUM/2020.4871) of the Abdul Wali Khan University Mardan. Written informed consent was obtained from the owners for the participation of their animals in this study.

## Author Contributions

HZ and AA designed the study and acquired the budget. HZ, AA, and MK, collected the samples. AA, HZ, SM-L, ASA, MK, and ML performed the experiments and analyzed the results. All authors performed critical revision and approved the final manuscript.

## Conflict of Interest

The authors declare that the research was conducted in the absence of any commercial or financial relationships that could be construed as a potential conflict of interest.
